# Temporal trends in management and outcome of pulmonary embolism: a single-centre experience

**DOI:** 10.1007/s00392-019-01489-9

**Published:** 2019-05-07

**Authors:** Matthias Ebner, Karl-Patrik Kresoja, Karsten Keller, Lukas Hobohm, Nina I. J. Rogge, Gerd Hasenfuß, Burkert Pieske, Stavros V. Konstantinides, Mareike Lankeit

**Affiliations:** 1 Department of Internal Medicine and Cardiology, Campus Virchow Klinikum, Charité-University Medicine Berlin, Augustenburger Platz 1, 13353 Berlin, Germany; 2 German Center for Cardiovascular Research (DZHK), Partner Site Berlin, Berlin, Germany; 3 Berlin Institute of Health (BIH), Berlin, Germany; 4 Center for Thrombosis and Hemostasis (CTH), University Medical Center Mainz, Mainz, Germany; 5 Cardiology I, Center for Cardiology, University Medical Center Mainz, Mainz, Germany; 6 Clinic of Cardiology and Pneumology, Heart Center, University Medical Center Goettingen, Goettingen, Germany; 7 German Center for Cardiovascular Research (DZHK), Partner Site Goettingen, Goettingen, Germany; 8 Department of Cardiology, Democritus University of Thrace, Alexandroupolis, Greece

**Keywords:** Pulmonary embolism, Risk assessment, Early discharge, Trends, Anticoagulation

## Abstract

**Background:**

Real-world data on the impact of advances in risk-adjusted management on the outcome of patients with pulmonary embolism (PE) are limited.

**Methods:**

To investigate temporal trends in treatment, in-hospital adverse outcomes and 1-year mortality, we analysed data from 605 patients [median age, 70 years (IQR 56–77) years, 53% female] consecutively enrolled in a single-centre registry between 09/2008 and 08/2016.

**Results:**

Over the 8-year period, more patients were classified to lower risk classes according to the European Society of Cardiology (ESC) 2014 guideline algorithm while the number of high-risk patients with out-of-hospital cardiac arrest (OHCA) increased. Although patients with OHCA had an exceptionally high in-hospital mortality rate of 59.3%, the rate of PE-related in-hospital adverse outcomes (12.2%) in the overall patient cohort remained stable over time. The rate of reperfusion treatment was 9.6% and tended to increase in high-risk patients. We observed a decrease in the median duration of in-hospital stay from 10 (IQR 6–14) to 7 (IQR 4–15) days, an increase of patients discharged early from 2.1 to 12.2% and an increase in the use of non-vitamin K-dependent oral anticoagulants (NOACs) from 12.6 to 57.2% in the last 2 years (09/2014–08/2016) compared to first 6 years (09/2008–08/2014). The 1-year mortality rate (16.9%) remained stable throughout the study period.

**Conclusion:**

In-hospital adverse outcomes and 1-year mortality remained stable despite more patients with OHCA, shorter in-hospital stays, more patients discharged early and a more frequent NOAC use.

**Electronic supplementary material:**

The online version of this article (10.1007/s00392-019-01489-9) contains supplementary material, which is available to authorized users.

## Introduction

Pulmonary embolism (PE) is a major contributor to global disease burden and associated with high morbidity and mortality [1-3]. Evidence published in the past decade consistently indicates an increase of diagnosed PE, most likely due to a higher number of patients at risk in aging societies and improvement of imaging techniques, in particular higher sensitivity of computed tomographic pulmonary angiography (CTPA) [4]. A meta-analysis including 22 studies demonstrated a duplication of the rate of subsegmental PE diagnosis in parallel with advances in CT technology, rising from 4.7 to 9.4% in patients undergoing single- and multi-detector CT, respectively [5]. Based on a retrospective analysis of US healthcare claims data, the annual prevalence of venous thromboembolism (VTE) is expected to double from 0.95 million in 2006 to 1.82 million in 2050 in the US [6].

Further, considerable progress in the management of acute PE was made over the past years [4]. The concept of risk-adjusted management was confirmed by randomised trials such as the pulmonary embolism thrombolysis (PEITHO) and the outpatient treatment of pulmonary embolism (OTPE) trials and management studies such as the Hestia study [7-9]. Evidence is accumulating that home treatment or early discharge is a valuable treatment option for selected patients with low-risk PE [10-12]. Thus, current guidelines emphasise the importance of risk stratification to guide risk-adjusted therapeutic decision-making [1, 13]. Additionally, non-vitamin K-dependent oral anticoagulants (NOACs) have emerged as a safe and efficacious alternative to vitamin K antagonists (VKAs) for therapeutic anticoagulation [1].

However, real-world data on the impact of advances in risk-adjusted management on patients’ outcome are limited. Two studies that investigated temporal trends in the management and outcome of PE patients provide only limited information on PE severity and the prognostic impact of risk assessment strategies [14, 15]. Furthermore, long-term mortality data were not provided and study inclusion periods ended in 2012 [14] and 2013 [15], respectively. Thus, the possible impact of the latest update of the European Society of Cardiology (ESC) guideline in 2014 [1] could not be explored.

Therefore, we evaluated changes in reperfusion treatment, length of hospital stays, in-hospital adverse outcomes and 1-year all-cause mortality in patients with acute PE included in a single-centre registry over an 8-year period to investigate temporal trends in risk-adjusted management and outcome of PE patients.

## Material and methods

### Study design and outcomes

The Pulmonary Embolism Registry of Goettingen (PERGO) prospectively includes consecutive patients with objectively confirmed PE ≥ 18 years of age admitted to the University Medical Center Goettingen, Germany. The study protocol has been described in detail previously [16, 17]. The present analysis included patients enrolled in PERGO between September 2008 and August 2016. Patients withdrawing previously given consent for participation in PERGO and patients included twice in PERGO because of recurrent PE were excluded from analysis. All patients were followed for the in-hospital stay and 1-year survival status was assessed by contacting the responsible registration offices.

Diagnostic and therapeutic management was in accordance with the ESC 2008 (09/2008–08/2014) and 2014 (09/2014–08/2016) guidelines [1, 18] and local standard operating procedures. All related decisions were left to the discretion of the treating physicians and were not influenced by the study protocol. Treating physicians were not informed about study results, thus any influence of the study on patient management or monitoring of treatment effects during the follow-up period can be excluded.

Complete data on baseline characteristics, VTE risk factors and comorbidities, results from diagnostic examinations including imaging (CTPA and transthoracic echocardiography) and laboratory testing, treatment and in-hospital outcomes were obtained using a standardised questionnaire case report form. Acute reperfusion treatment was defined as systemic thrombolysis, surgical thrombectomy and interventional approaches. Early discharge was defined as discharge from hospital within 48 h. Further definitions used in the present study are provided in the Online Resource. Patients were classified to risk classes according to the algorithm proposed by the ESC 2014 guidelines [1], the simplified Pulmonary Embolism Severity Index (sPESI), the modified FAST score [19] and the Bova score [20]. For calculation of algorithms and scores, missing values were considered to be normal [19].

An in-hospital adverse outcome was defined as PE-related death, need for mechanical ventilation, cardiopulmonary resuscitation or administration of catecholamines. Further study outcomes include in-hospital all-cause death, duration of the in-hospital stay (days) and 1-year all-cause mortality. Death was determined to be PE-related if either confirmed by autopsy or following a clinically severe episode of acute PE in absence of an alternative diagnosis. All events and causes of death were independently adjudicated by two of the authors (M.E. and K.K.) and disagreement was resolved by a third author (M.L.).

### Statistical analysis

Categorical variables are presented as total numbers and percentages, continuous variables not following a normal distribution if tested with Kolmogorov–Smirnov test are presented as medians with interquartile ranges (IQR). Associations between binary and categorical variables were analysed using Fisher’s exact test or Chi-squared test, as appropriate. For comparison of continuous variables, the Mann–Whitney *U* test was employed.

For analysis of temporal trends, the study period was divided in four 2-year segments (09/2008–08/2010; 09/2010–08/2012; 09/2012–08/2014; 09/2014–08/2016). Testing for temporal trends was conducted using the Cochran–Armitage trend test for binary variables, the Mantel–Haenszel test of trend for categorial variables and linear regression for continuous variables.

Receiver operating characteristic (ROC) curve analyses were performed to determine the area under the curve (AUC) of risk scores with regard to study outcomes. Comparison of the prognostic performance of dichotomous algorithms and scores was performed by calculation of sensitivity, specificity, positive/negative predictive value and positive/negative likelihood ratios. To allow comparison of scores, the three-level ESC 2014 algorithm and Bova score were dichotomised as low- and intermediate-low risk (“low-risk”) versus intermediate-high risk (“intermediate-high risk”) [19]. The prognostic relevance of dichotomous algorithms and scores as well as single predictors with regard to study outcomes was tested using univariable logistic regression analyses and results are presented as odds ratios (OR) with the corresponding 95% confidence intervals (CIs). Kaplan–Meier analysis was used to compare the probability of 1-year survival in subgroups stratified according the ESC risk classes; the log-rank test was used for comparison.

A two-sided significance level of *α* < 0.05 was defined appropriate to indicate statistical significance. As this was an explorative testing, no adjustments for multiple testing were carried out. *p* values were provided for descriptive reasons only and should be interpreted with caution and in connection with effect estimates. Statistical analysis was performed through Statistics Package for Social Sciences (IBM SPSS Statistics, Version 23, IBM Corp. Armonk, NY, USA).

## Results

### Trends in initial presentation and risk stratification

Over the 8-year study period, 605 patients were included in the present analysis. Diagnostic procedures are described in the Online Resource and Table 1s. The clinical characteristics, comorbidities and initial presentation of study patients are shown in Table 1, left column. By dividing the observation period into four 2-year segments, differences in the prevalence of risk factors and comorbidities over time were observed (Table 1, right columns): while the proportion of patients with immobilisation or chronic heart failure decreased (*p* < 0.001 and *p* = 0.001 for trend, respectively), an increase of patients who underwent surgery within 4 weeks prior to PE (*p* = 0.012 for trend) was observed.

**Table 1:** Baseline characteristics, comorbidities and initial presentation of study patients stratified according to time intervals

Observation period	09/2008–08/2016 (*n* = 605)	09/2008–08/2010 (*n* = 145)	09/2010–08/2012 (*n* = 140)	09/2012–08/2014 (*n* = 165)	09/2014–08/2016 (*n* = 155)	*p* for trend
Age (years)	70 [56–77]	70 [55–76]	71 [55–77]	69 [57–78]	67 [55–77]	0.94
Sex (female)	320/605 (52.9%)	77/145 (53.1%)	78/140 (55.7%)	87/165 (52.7%)	78/155 (50.3%)	0.53
BMI (kg/m^2^)	27.6 [24.3–31.2]	27.1 [23.9–31.0]	27.8 [24.1–30.5]	27.8 [25.0–33.1]	26.8 [24.2–31.1]	0.23
**Risk factors for VTE and comorbidities**						
Previous VTE	161/603 (26.6%)	37/145 (25.5%)	40/140 (28.6%)	53/163 (32.5%)	31/155 (20%)	0.43
Surgery (previous 4 weeks)	109/605 (18.0%)	20/145 (13.8%)	19/140 (13.6%)	34/165 (20.6%)	36/155 (23.2%)	0.012
Trauma (previous 4 weeks)	18/605 (3.0%)	5/145 (3.4%)	5/140 (3.6%)	2/165 (1.2%)	6/155 (3.9%)	0.86
Immobilisation (previous 4 weeks)	128/604 (21.2%)	54/145 (37.2%)	25/139 (18.0%)	20/165 (12.1%)	28/155 (18.1%)	< 0.001
Travel	31/603 (5.1%)	9/145 (6.2%)	7/139 (5.0%)	5/164 (3.0%)	10/155 (6.5%)	0.88
Active cancer	102/605 (16.9%)	27/145 (18.6%)	22/140 (15.7%)	26/165 (15.8%)	27/155 (17.4%)	0.80
Chronic heart failure	96/605 (15.9%)	32/145 (22.1%)	27/140 (19.3%)	23/165 (13.9%)	14/155 (9.0%)	0.001
Chronic pulmonary disease	93/605 (15.7%)	20/145 (13.8%)	23/140 (16.4%)	22/165 (13.3%)	28/155 (18.1%)	0.46
Renal insufficiency	207/589 (35.1%)	39/144 (27.1%)	57/139 (41.0%)	64/161 (39.8%)	47/145 (32.4%)	0.39
**Symptoms and clinical findings**						
Chest pain	292/605 (48.5%)	71/145 (49.0%)	72/138 (52.2%)	90/165 (54.5%)	59/154 (38.3%)	0.11
Dyspnoea	496/605 (82.4%)	112/145 (77.2%)	125/138 (90.6%)	147/165 (89.1%)	112/154 (72.7%)	0.28
Haemoptysis	18/605 (3.0%)	5/145 (3.4%)	3/139 (2.2%)	6/165 (3.6%)	4/154 (2.6%)	0.87
Syncope	94/604 (15.6%)	30/145 (20.7%)	22/140 (15.7%)	24/165 (14.5%)	18/154 (11.7%)	0.034
Unilateral leg swelling	138/600 (23.0%)	46/144 (31.9%)	38/137 (27.7%)	29/165 (17.6%)	25/154 (16.2%)	< 0.001
Tachycardia	208/581 (32.5%)	47/143 (30.8%)	54/138 (37.7%)	56/156 (31.4%)	51/144 (30.6%)	0.80
Hypotension	34/570 (6.0%)	10/142 (7.0%)	10/137 (7.3%)	10/154 (6.5%)	4/137 (2.9%)	0.15
Hypoxia	147/511 (28.7%)	32/116 (27.6%)	36/120 (21.7%)	51/146 (34.9%)	28/129 (21.7%)	0.49
RV dysfunction on TTE/CT	284/549 (51.7%)	70/134 (52.2%)	83/139 (59.7%)	74/159 (46.5%)	57/117 (48.7%)	0.21
Elevated troponin	359/531 (67.6%)	92/127 (72.4%)	88/133 (66.2%)	92/145 (63.4%)	87/126 (69.0%)	0.47
Elevated NT-proBNP	263/460 (57%)	78/132 (59.1%)	79/127 (62.2%)	53/96 (55.2%)	53/105 (50.5%)	0.40

*BMI* body mass index, *VTE* venous thromboembolism, *RV* right ventricular, *TTE* transthoracic echocardiography, *CT* computed tomography, *NT-proBNP* N-terminal pro-brain natriuretic peptide

**Fig. 1:**
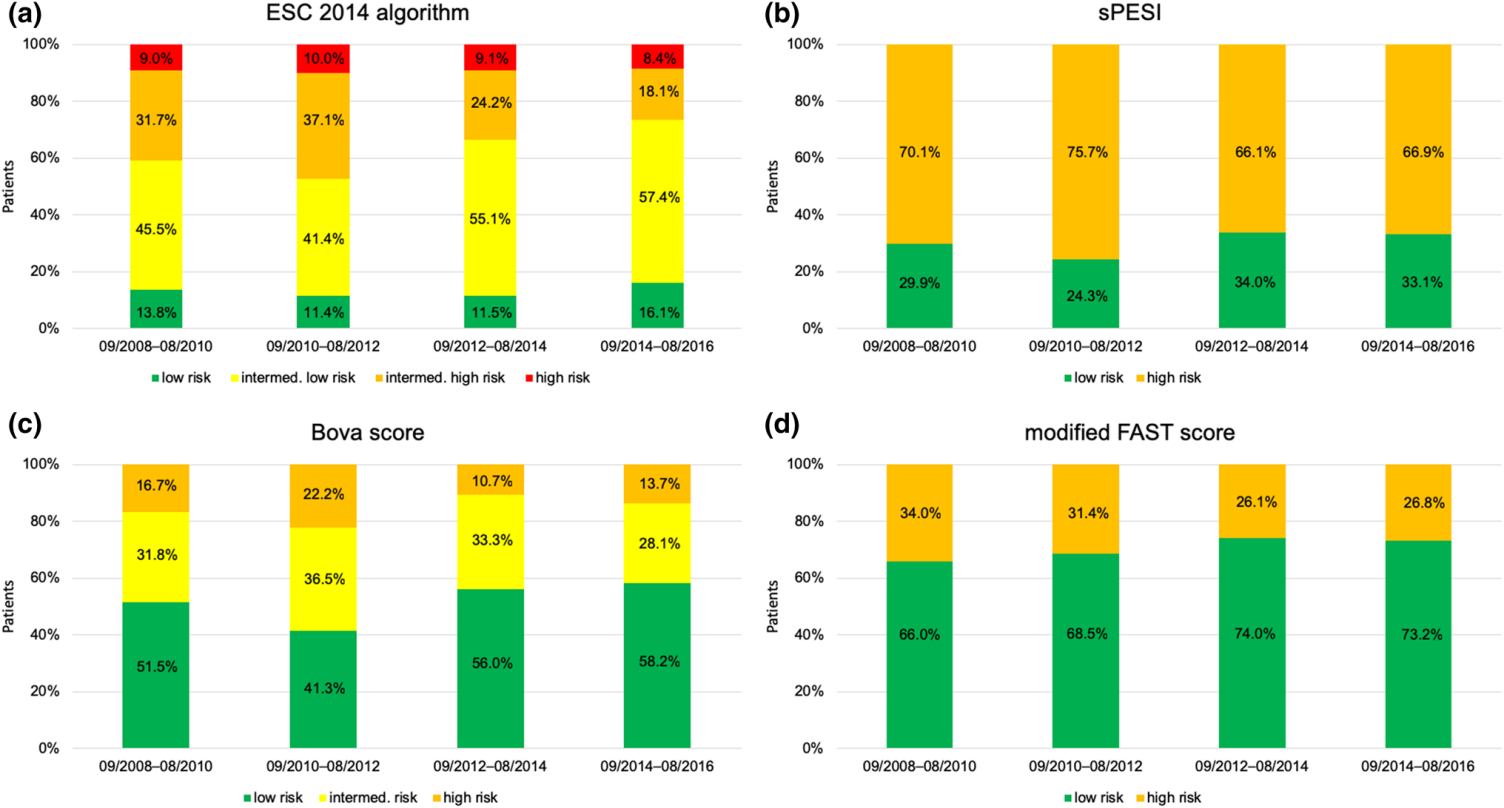
Stratification of PE patients into risk classes over time. Patients were stratified in risk classes according to ESC 2014 algorithm (**a**), sPESI (**b**), Bova score (**c**) and modified FAST score (**d**). *ESC* European Society of Cardiology, *sPESI* simplified pulmonary embolism severity index

Overall, 80 (13.2%) patients were classified as low risk, 304 (50.2%) as intermediate-low risk, 166 (27.4%) as intermediate-high risk and 55 (9.1%) as high risk according to the algorithm for risk stratification proposed by the ESC 2014 guideline. In high-risk patients, out-of-hospital cardiac arrest (OHCA) occurred in 27 (49.1%) patients. Throughout the study period, more patients were classified to low- and intermediate-low risk classes using the ESC 2014 algorithm (*p* = 0.026 for trend, Fig. 1a). A similar temporal trend was observed for the Bova score (*p* = 0.046 for trend), while risk stratification using the sPESI and the modified FAST score showed no changes over time (Fig. 1b–d).

### Trends in in-hospital outcomes

**Fig. 2:**
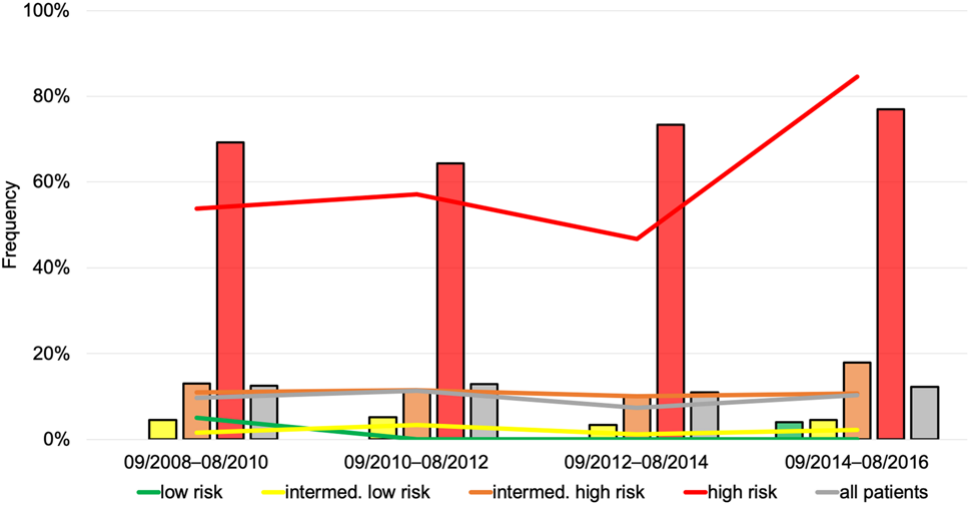
Rates of acute reperfusion and an in-hospital adverse outcome over time. Rates of acute reperfusion treatment and in-hospital adverse outcome stratified according to the ESC 2014 algorithm. Lines represent the percentage of patients who underwent reperfusion treatment, bars represent the percentage of patients with an in-hospital adverse outcome

**Fig. 3:**
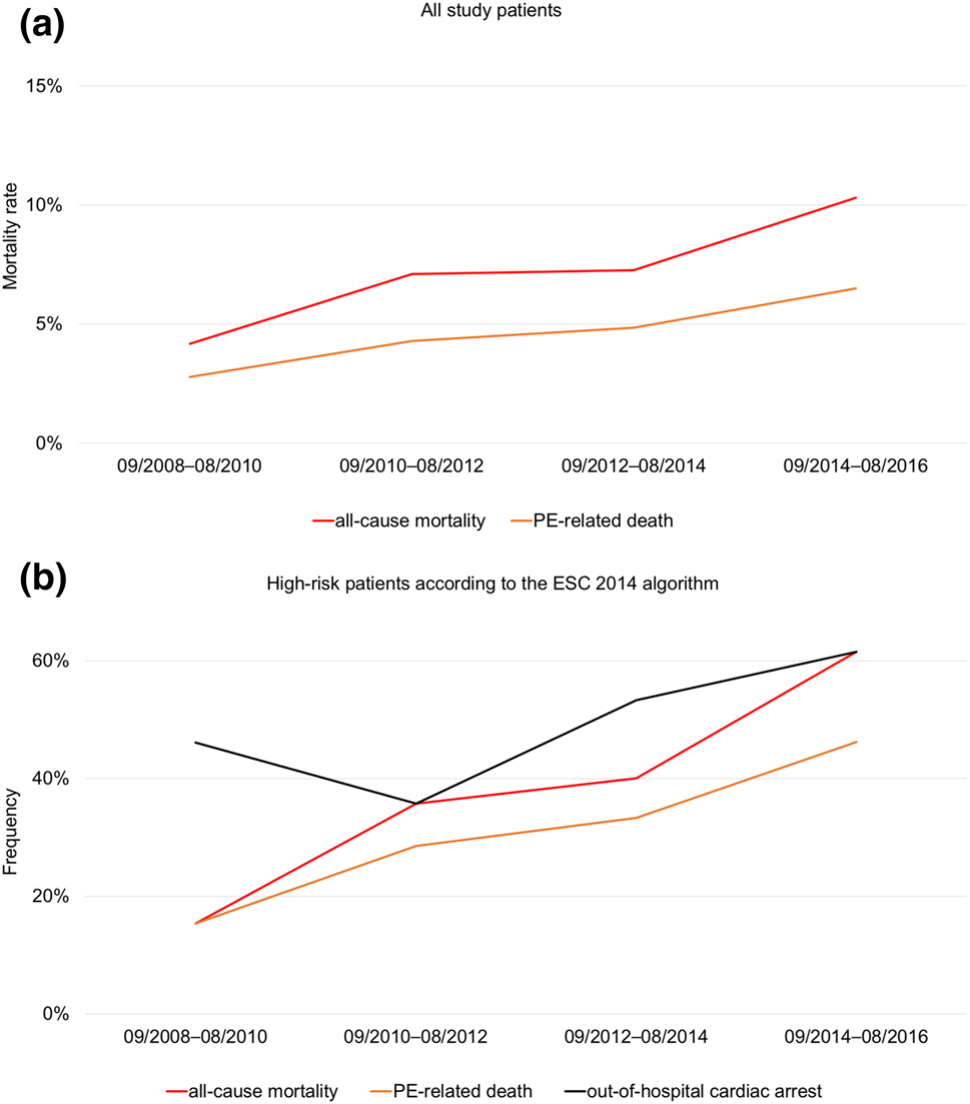
Rates of in-hospital mortality over time. Rates of in-hospital all-cause and PE-related mortality in all study patients (**a**) and in high-risk patients (**b**). Additionally, in **b**, the proportion of high-risk patients with out-of-hospital cardiac arrest is shown (black line)

Overall, 74 (12.2%) patients had an in-hospital adverse outcome. As shown in Table 2 and Fig. 2, no differences in the rate of an in-hospital adverse outcome were observed over time (*p* = 0.97 for trend). Forty-four (7.3%) patients died during the in-hospital stay [median time to death, 8 (2–15) days]; 66% of deaths were related to the acute PE. Interestingly, the in-hospital mortality rate increased from 4.1% in the first 2-year segment to 10.3% in the last 2-year segment (*p* = 0.049 for trend, Table 2 and Fig. 3a) paralleled by an increase of high-risk PE patients presenting with OHCA (Fig. 3b). Of note, the increase in mortality was considerably attenuated if OHCA patients were excluded from the analysis (3.6–6.2% from the first to the last 2-year segment, *p* = 0.34 for trend).

**Table 2:** Temporal changes in in-hospital adverse outcomes and 1-year all-cause mortality

Observation period	09/2008–08/2016	09/2008–08/2010	09/2010–08/2012	09/2012–08/2014	09/2014–08/2016	*p* for trend
**A: In-hospital adverse outcome**	74/605 (12.2%)	18/145 (12.4%)	18/140 (12.9%)	18/165 (10.9%)	20/155 (12.9%)	0.97
Low risk	1/80 (1.3%)	0/20 (0.0%)	0/16 (0.0%)	0/19 (0.0%)	1/25 (4.0%)	0.24
Intermediate-low risk	13/304 (4.3%)	3/66 (4.5%)	3/58 (5.2%)	3/91 (3.3%)	4/89 (4.5%)	0.86
Intermediate-high risk	21/166 (12.7%)	6/46 (13.0%)	6/52 (11.5%)	4/40 (10%)	5/28 (17.9%)	0.71
High-risk	39/55 (70.9%)	9/13 (69.2%)	9/14 (64.3%)	11/15 (73.3%)	10/13 (76.9%)	0.56
**B: In-hospital all-cause mortality**	44/605 (7.3%)	6/145 (4.1%)	10/140 (7.1%)	12/165 (7.3%)	16/155 (10.3%)	0.049
Low risk	1/80 (1.3%)	0/20 (0.0%)	0/16 (0.0%)	0/19 (0.0%)	1/25 (4.0%)	0.24
Intermediate-low risk	10/304 (3.3%)	1/66 (1.5%)	3/58 (5.2%)	3/91 (3.3%)	3/89 (3.4%)	0.70
Intermediate-high risk	12/166 (7.2%)	3/46 (6.5%)	2/52 (3.8%)	3/40 (7.5%)	4/28 (14.3%)	0.21
High-risk	21/55 (38.2%)	2/13 (15.4%)	5/14 (35.7%)	6/15 (40.0%)	8/13 (61.5%)	0.019
**C: Death after discharge**	58/561 (10.3%)	20/139 (14.4%)	12/130 (9.2%)	15/153 (9.8%)	11/139 (7.9%)	0.10
Low risk	0/79 (0%)	0/20 (0.0%)	0/16 (0.0%)	0/19 (0.0%)	0/24 (0.0%)	n.c
Intermediate-low risk	29/294 (9.8%)	7/65 (10.8%)	4/55 (7.3%)	11/88 (12.5%)	7/86 (8.1%)	0.83
Intermediate-high risk	21/154 (13.6%)	8/43 (18.6%)	6/50 (12.0%)	4/37 (10.8%)	3/24 (12.5%)	0.40
High-risk	8/34 (23.5%)	5/11 (45.5%)	2/9 (22.2%)	0/9 (0.0%)	1/5 (20.0%)	0.07
**D: Overall 1-year mortality**	102/605 (16.9%)	26/145 (17.9%)	22/140 (15.7%)	27/165 (16.4%)	27/155 (17.4%)	0.95
Low risk	1/80 (1.3%)	0/20 (0.0%)	0/16 (0.0%)	0/19 (0.0%)	1/25 (4.0%)	0.24
Intermediate-low risk	39/304 (12.8%)	8/66 (12.1%)	7/58 (12.1%)	14/91 (15.4%)	10/89 (11.2%)	1.00
Intermediate-high risk	33/166 (19.9%)	11/46 (23.9%)	8/52 (15.4%)	7/40 (17.5%)	7/28 (25.0%)	0.99
High-risk	29/55 (52.7%)	7/13 (53.8%)	7/14 (50.0%)	6/15 (40.0%)	9/13 (69.2%)	0.58

Patients were stratified in risk classes according to ESC 2014 algorithm

High-risk patients had the highest rate of an in-hospital adverse outcome (70.9%) and in-hospital all-cause mortality (38.2%). OHCA patients had a substantially higher in-hospital all-cause mortality rate compared to patients without OHCA (59.3% vs. 4.7%, risk ratio 12.7, *p* < 0.001).

In not-high-risk patients, 6.4% had an in-hospital adverse outcome and 4.2% died; of those, 47.8% died of PE. Information on the overall prognostic performance of different risk assessment strategies are provided in the Online Resource Table 2s. Between the first and the second half of the study period, no differences in the prognostic performance of cardiac biomarkers, the modified FAST score or the ESC 2014 algorithm were observed (Table 3s of the Online Resource).

### Trends in acute reperfusion treatment and impact on in-hospital outcomes

Reperfusion treatment was performed in 58 (9.6%) patients; details are provided in the Online Resource. Briefly, 55 patients received intravenous systemic thrombolysis and five patients underwent surgical thrombectomy, alone or in combination. Rates of reperfusion treatment remained stable throughout the study period (Fig. 2; *p* = 0.64 for trend).

Overall, 60.0% of all patients classified as high risk according the ESC 2014 algorithm were treated with reperfusion therapy. High-risk patients receiving reperfusion treatment more often had RV dysfunction (87.5% vs. 57.8%, *p* = 0.038) and OHCA (60.6% vs. 31.8%, *p* = 0.036) compared to those not receiving reperfusion therapy. As shown in Fig. 2, there was a trend towards a higher reperfusion rate in high-risk patients after publication of the ESC 2014 guideline. In intermediate–high risk patients, 10.2% received systemic thrombolysis and 16.9% were enrolled in the PEITHO trial.

In high-risk patients receiving reperfusion treatment a higher in-hospital adverse outcome rate was observed compared to patients not treated with reperfusion therapy (84.8% vs. 50.0%, *p* = 0.005). Adverse events during the in-hospital stay according to the treatment performed are provided in Table 3. Major bleeding occurred more often in patients treated with thrombolysis or included in the PEITHO trial compared to patients treated with anticoagulation only (22.8% and 8.8% vs. 3.7%, *p* < 0.001 and *p* = 0.14, respectively). Thrombolysis was associated with an 8.0-fold increased risk for major bleeding (95% CI 3.7–17.4; *p* < 0.001). Rates of major bleeding remained stable throughout the study period (*p* = 0.12 for trend); only one patient had fatal bleeding.

**Table 3:** Frequency of in-hospital adverse events according to treatment performed

	Adverse outcome	Resuscitation	Catecholamines	Mechanical ventilation	PE-related death	All-cause death	Major bleeding^a^
Anticoagulation only	33/515 (6.4%)	8/515 (1.6%)	22/515 (4.3%)	21/514 (4.1%)	12/515 (2.3%)	23/515 (4.5%)	21/515 (4.1%)
Thrombolysis or thrombectomy	37/56 (66.1%)	21/56 (37.5%)	32/56 (57.1%)	27/56 (48.2%)	17/56 (30.4%)	20/56 (35.7%)	13/56 (23.2%)
Inclusion in PEITHO	4/34 (11.8%)	1/34 (2.9%)	4/34 (11.8%)	3/34 (8.8%)	0/34 (0%)	1/34 (2.9%)	3/34 (8.8%)
All patients	74/605 (12.2%)	30/605 (5.0%)	58/605 (9.6%)	51/605 (8.4%)	29/605 (4.8%)	44/605 (7.3%)	37/605 (6.1%)

*PE* pulmonary embolism, *PEITHO* pulmonary embolism thrombolysis trial

^a^One patient died due to fatal bleeding

### Trends in length of in-hospital stay and anticoagulant management at discharge

**Fig. 4:**
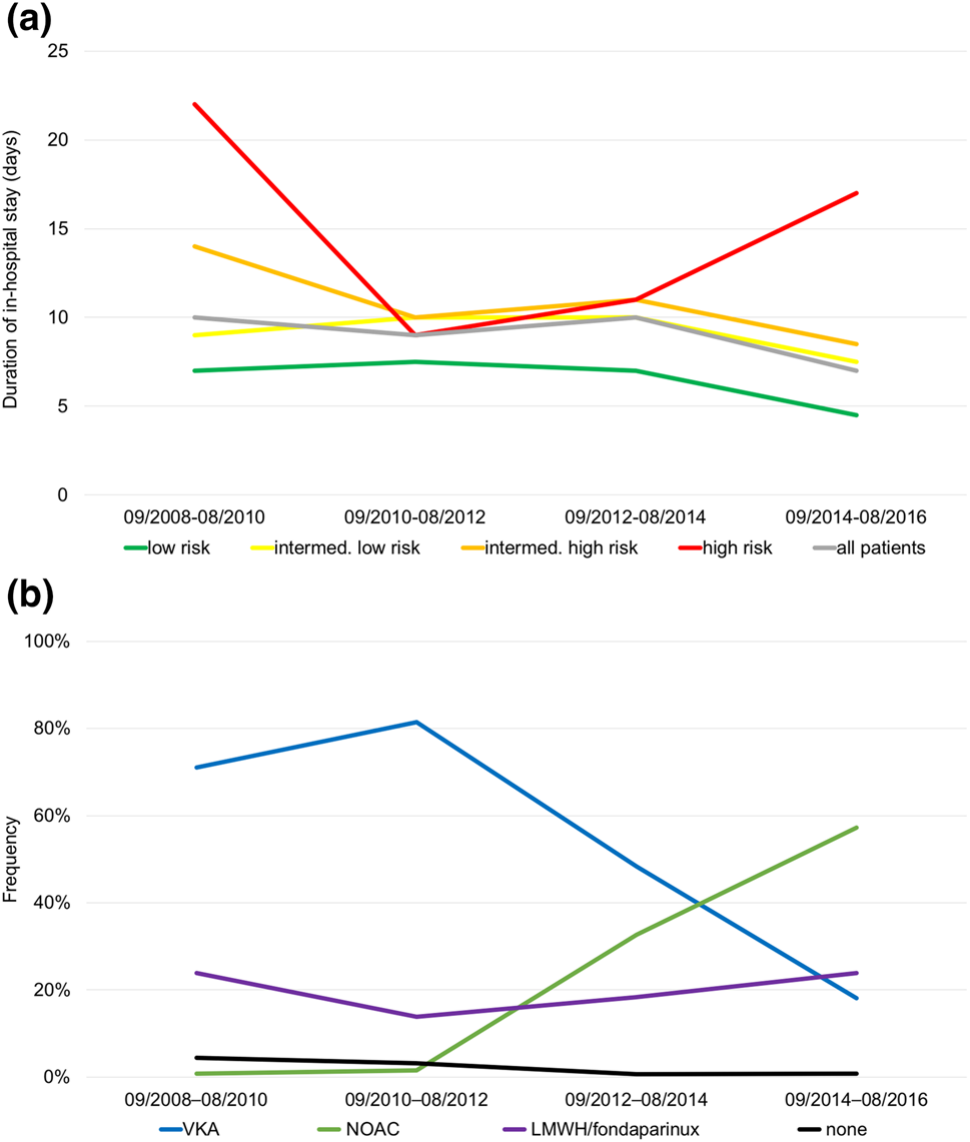
Median duration of in-hospital stays (**a**) and therapeutic anticoagulation at discharge (**b**) over time. In **a**, patients were stratified in risk classes according to ESC 2014 algorithm. *VKA* vitamin K antagonists, *NOAC* non-vitamin K oral anticoagulants, *LMWH* low molecular weight heparin

As shown in Fig. 4a, the median duration of in-hospital stays of patients who were discharged alive remained stable during the first 6 years of the study period [09/2008–09/2010: 10 (IQR 7–16) days, 09/2010–09/2012: 9 (IQR 6–14) days, 09/2012–09/2014: 10 (IQR 7–15) days]. However, there was a decrease of the median duration of in-hospital stay in the last 2-year segment [09/2014–08/2016: 7 (IQR 4–15) days, *p* = 0.01 compared to 09/2008–08/2014]. Furthermore, the proportion of patients discharged early increased from 5.0% in the first 2-year segment to 12.2% in the last 2-year segment (*p* = 0.009 for trend).

Of 561 patients (92.7%) discharged alive, 547 patients (97.5%) were treated with therapeutic anticoagulation: 303 (54.0%) were treated with VKAs, 132 (23.5%) with NOACs and 112 (20.0%) with low molecular weight heparin (LMWH)/Fondaparinux. Twelve (2.1%) were discharged without therapeutic anticoagulation, most often due to exceptionally high bleeding risk or refusal by the patient. Information was missing for two patients (0.4%). As expected, treatment with NOACs increased over time (*p* < 0.001 for trend; Fig. 4b); for example, 86.7% of patients discharged early were treated with NOACs in the last 2-year segment.

### Trends in 1-year mortality

**Fig. 5:**
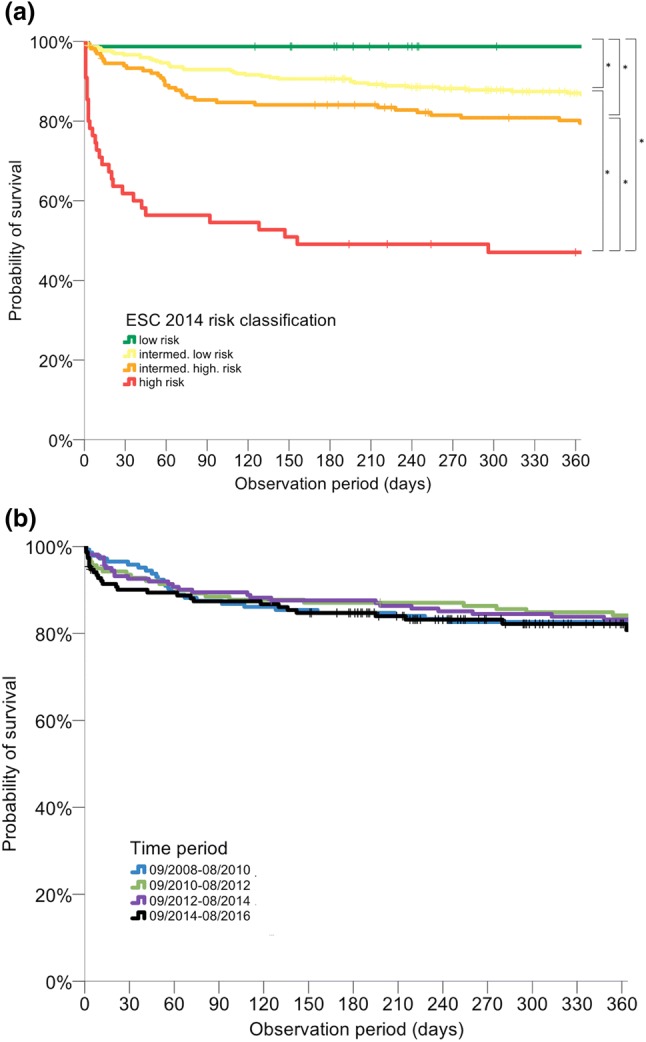
Probability of 1-year all-cause mortality. 1-year all-cause mortality rate stratified according to the ESC 2014 algorithm (**a**) and inclusion period (**b**). Stars signify statistically significant differences between risk groups

Information on 1-year survival status with a complete follow-up time of 365 days was available for 592 (97.9%) patients. Within 1 year after PE, 102 (16.9%) patients died [median time-to-death 50 (IQR 10–126) days]. Baseline characteristics of patients who died in-hospital (*n* = 44) and of patients who died after discharge (*n* = 58) are compared to those of 1-year survivors (*n* = 503) in Table 4s of the Online Resource. The prognostic value of risk markers and assessment strategies with regard to in-hospital and 1-year all-cause mortality is shown in Table 5s of the Online Resource. The 1-year mortality rates ranged from 1.3% in patients classified as low-risk according the ESC 2014 algorithm to as high as 52.7% in high-risk patients (Table 2 and Fig. 5a) and did not differ between patients discharged with VKA compared to patients discharged with NOAC (6.3% vs. 5.5%, *p* = 0.81). Of patients discharged alive from hospital, no patient classified as low-risk according to the ESC 2014 algorithm died within 1 year after PE, while high-risk patients remained at elevated risk of 1-year mortality (OR, 2.9; 95% CI 1.3–6.8; Table 5s). No temporal changes in 1-year mortality rates were found throughout the study period (*p* = 0.95 for trend; Table 2 and Fig. 5b).

## Discussion

In the present real-world single-centre cohort investigating 605 PE patients, more patients were classified to lower risk classes according to the algorithm proposed by the ESC 2014 guideline over the 8-year study period. Despite an increase of patients with OHCA, a subgroup with exceptional high mortality, the rate of PE-related in-hospital adverse outcomes did not change over time. Reperfusion treatment was administered in almost 10% of patients (and in more than 60% of high-risk patients) and its use tended to increase in the last 2-year segment. At the same time, NOACs became the most frequently used anticoagulant agents at discharge and a reduction of the median length of in-hospital stay was observed. The 1-year all-cause mortality rate was high and remained stable over time.

### Patient cohort

We report on a large and well characterised population of consecutive PE patients from a tertiary referral university centre. In contrast, previous publications investigating trends in PE were based on a discharge database [14] and non-consecutive multicentric registry data [15]. Thus, detailed information on patients’ baseline characteristics including VTE risk factors and comorbidities, results of diagnostic examinations including imaging and laboratory testing, risk prediction scores, treatment and outcome were available. Since information on RV (dys)function and troponin concentrations were available for almost all study patients (90.7% and 87.8%, respectively), the proportion of “low-risk” patients (defined by a sPESI of 0 points plus absence of RV dysfunction on imaging plus normal troponin levels) was lower compared to other registries or cohort studies defining “low-risk” PE based on a sPESI of 0 points alone. Although more patients were classified to lower risk classes according to the ESC 2014 algorithm over time, the overall proportion of high-risk patients (9.1%, Fig. 1) remained stable and was larger compared to other registries: for example, only 58 of 1880 patients (3.0%) of the US Multicenter Emergency Medicine Pulmonary Embolism in the Real World Registry (EMPEROR, inclusion period 2005–2008) and only 778 of 23,858 patients (3.3%) of the Registro Informatizado de la Enfermedad TromboEmbólica (RIETE, inclusion period 2001–2013) had a systolic blood pressure < 90 mmHg at presentation [15, 21]. In contrast, in a more recent pooled analysis of three prospective European cohorts (inclusion period 2010–2014), 105 of 906 patients (11.6%) presented with high-risk PE [22]. However, it remains speculative whether the higher prevalence of high-risk PE patients in the present cohort may be attributable to the study design and/or advances in health and emergency systems resulting in more critically ill patients reaching the hospital. The latter hypothesis may be supported by the observed increase of patients with OHCA over time in the present study.

### Trends in in-hospital outcome

In the present single-centre cohort, 12.2% patients had an in-hospital adverse outcome and 7.3% died during the in-hospital stay. The most relevant contributor to adverse outcomes was a small subgroup of patients classified as high-risk according to the ESC 2014 algorithm who received reperfusion therapy or presented with OHCA: these 42 (6.9%) patients accounted for 46.5% of all adverse events. Congruently, high-risk patients had a higher in-hospital mortality rate (38.2%) compared to 22.0% reported by Becattini and colleagues [22]. In contrast, in low- and intermediate-low risk patients of the present single-centre cohort, a lower in-hospital mortality rate (1.3% and 3.3%, respectively, Table 2 and Fig. 2) compared to the findings from Becattini and colleagues (0.5% and 6.0%, respectively [22]) and a lower rate of in-hospital adverse outcomes (1.3% and 4.3%, respectively, Table 2 and Fig. 2) compared to 848 normotensive PE patients of the PROTECT study (1.6% and 10.0%, respectively [23]) was observed.

However, temporal analyses of our data revealed an increase in all-cause in-hospital mortality contrary to previous studies investigating trends of mortality in PE (reviewed and summarised in [4]). For example, in 60,853 patients diagnosed with PE in northwestern Italy between 2002 and 2012, the case fatality rate decreased throughout the study period (from 15.6% in 2002 to 10.2% in 2012 in women and from 17.6% in 2002 to 10.1% in 2012 in men) [14]. In 23,858 PE patients from RIETE, the risk-adjusted rates of all-cause mortality decreased from 6.5% in the first period (2001–2005) to 4.9% in the last period (2010–2013) [15]. Of note, in the present study, the increase of the in-hospital mortality rate only affected the subgroup of high-risk PE patients (Table 2) and was paralleled by a higher number of patients presenting with OHCA (Fig. 3b). Exclusion of OHCA patients from analysis considerably attenuated this trend. The rate of in-hospital adverse outcomes did not change over time (Fig. 2).

### Trends in risk-adjusted management

Since our study included PE patients enrolled until August 2016, we were able to provide information on the possible impact of recommendation related to risk-adjusted management introduced with the ESC 2014 guidelines. Reperfusion therapy was employed at an overall high rate of 9.6% over the study period, compared to less than 2% in RIETE [15]. In our high-risk patients, systemic thrombolytic therapy was performed in 60.0% of patients, a rate considerably higher than the 29.6% reported based on data from the US Nationwide Inpatient Sample [24]. Interestingly, and in contrast to findings from RIETE covering an observation period from 2001 to 2013 [15], our data indicated a trend towards an increased use of reperfusion therapy in high-risk patients after publication of the ESC 2014 guidelines. As expected, patients who received thrombolytic therapy had a higher incidence of major bleeding and thrombolysis was associated with an 8.0-fold increased risk of in-hospital major bleeding. Noteworthy, high-risk patients who did not receive reperfusion treatment had a lower in-hospital mortality rate compared to high-risk patients treated with reperfusion therapy, paralleled by a lower prevalence of RV dysfunction on TTE/CT and OHCA in the former patients. This observation might suggest that the current definition of “high-risk” PE of the ESC 2014 guideline [1], which allows classification of patients as “high-risk” based on systolic blood pressure only, may incorrectly classify a subgroup of patients as haemodynamically unstable who are not truly at “high risk” of an PE-related adverse outcome.

Of note, although no patient was treated with interventional approaches, percutaneous catheter-directed treatment (and especially ultrasound-based thrombus fragmentation combined with low-dose local thrombolysis) might constitute a novel and promising treatment option for selected (intermediate-high risk) patients [25]. Furthermore, veno-arterial extracorporeal membrane oxygenation (ECMO) was only used in a very limited number of patients. Increasing availability of mechanical cardiopulmonary support systems might improve the prognosis of high-risk patients with cardiac shock (although currently available data indicate a benefit of ECMO only as a complement to surgical embolectomy [26]).

NOACs became available during the second half of our study period (09/2012–08/2016) and were rapidly adopted as the preferential anticoagulation strategy, representing the most frequently prescribed anticoagulant agents in the last 2-year segment (Fig. 4b). This finding is in accordance with data from the US IMS Health National Disease and Therapeutic Index reporting a growth of NOAC use to 36% of VTE outpatient treatment visits in 2014 [27].

Finally, and in accordance with data from RIETE demonstrating a relative reduction of the mean length of in-hospital stay of 32% from 13.6 to 9.3 days [15], a similar relative reduction of 30% in the median duration of in-hospital stay from 10 days in the first 2-year segment (09/2008–09/2010) to 7 days in the last 2-year segment (09/2014–08/2016) was observed in the present cohort (Fig. 4a). While the length of hospitalisation remained stable over the first 6 years of the study period, the decrease after August 2014 might on the one hand be attributable to the introduction of the ESC 2014 guideline that strengthened the option of early discharge for selected low-risk patients [1]. Indeed, the proportion of patients discharged early increased to 12.2% in the last 2-year segment (09/2014–08/2016) compared to 2.1% in the first 6 years (09/2008–08/2014). On the other hand, the more frequent use of NOACs might have contributed to a shorter duration of in-hospital stay in the present real-world cohort. In accordance, a pooled analysis of the EINSTEIN-DVT and EINSTEIN-PE studies demonstrated a shorter length of in-hospital stay in rivaroxaban-treated patients compared to patients treated with enoxaparin/warfarin [28].

### Trends in 1-year mortality

To investigate the impact of temporal changes in risk-adjusted management on long-term mortality, study patients were followed for 1 year. The overall 1-year mortality rate was high (16.9%) and similar to ones reported for patients with acute decompensated heart failure, acute myocardial infarction or stroke [29, 30]. Although patients who died after hospital discharge more frequently were diagnosed with cancer compared to overall survivors (Table 4s), patients classified as high-risk according the ESC 2014 algorithm or with elevation of troponin or NT-proBNP plasma concentrations were at elevated risk to die within 1 year after PE, even if discharged alive (Table 5s). Thus, risk stratification appears not only to provide guidance for the management during the acute phase but may also help to identify patients with a higher risk of mortality in the first year after PE. Despite an increase of in-hospital mortality (presumable due to a higher rate of patients with OHCA, as discussed above), the 1-year mortality rate remained stable over time (Table 2 and Fig. 5b). In particular, neither the shorter duration of in-hospital stays and increase of patients discharged early nor the more frequent use of NOACs negatively affect 1-year survival.

### Limitations

The low patient and event numbers, especially if the patient cohort was stratified according to risk classes and observation period time segments, constitutes a potential limitation of our study. Further, the single-centre design limits the generalisability of study findings.

## Conclusion

In the present real-world single-centre cohort of 605 PE patients included over an 8-year period, the rates of in-hospital adverse outcomes remained stable despite an increase of patients with OHCA. Reperfusion treatment was administered in every 10th patient and its use tended to increase in high-risk patients. The implementation of the ESC 2014 guidelines and a more frequent use of NOACs might have contributed to the reduction of the length of in-hospital stay and a higher number of patients discharged early. The results of 1-year mortality analyses did not raise concerns regarding the safety of these approaches although 1-year mortality rates remained substantially high.

## Electronic supplementary material

Below is the link to the electronic supplementary material.

Supplementary file1 (DOCX 61 kb)
