# Supplementary material for: Temporal trends in management and outcome of pulmonary embolism: a single-centre experience
Source: Clin Res Cardiol. 2019 May 7;109(1):67–77. doi: 10.1007/s00392-019-01489-9 (PMC6952327; doi:10.1007/s00392-019-01489-9)
Supplement: Supplementary file 1 — Supplementary file1 (DOCX 61 kb) [file 392_2019_1489_MOESM1_ESM.docx]

**Online Resource**

**Temporal trends in management and outcome of pulmonary embolism:** **a single centre experience**

Matthias Ebner, Karl-Patrik Kresoja, Karsten Keller, Lukas Hobohm, Nina I. J. Rogge, Gerd Hasenfuß, Burkert Pieske, Stavros V. Konstantinides, Mareike Lankeit

**Published in:**

Clinical Research in Cardiology

**Corresponding author:**

Mareike Lankeit, MD, PhD, FESC, Department of Internal Medicine and Cardiology, Campus Virchow Klinikum, Charité – University Medicine Berlin, Augustenburger Platz 1, 13353 Berlin, Germany. Phone: +49 (0)30 / 450 665 381, fax: +49 (0)30 / 450 7 565 381, email: [mareike.lankeit@charite.de](mailto:mareike.lankeit@charite.de)

**Supplementary Material and Methods**

*Definitions*

Elevated troponin plasma concentrations were defined as high sensitivity troponin T (hsTnT) ≥14 pg/ml [1] or cardiac troponin I (cTnI) ≥13 ng/ml in female and ≥33 ng/ml in male patients. Elevated N-terminal pro-brain natriuretic peptide plasma (NT-proBNP) plasma concentrations were defined as ≥600 pg/ml [2].

Right ventricular (RV) dysfunction on diagnostic computed tomographic pulmonary angiography (CTPA) was defined as right-to-left (RV/LV) diameter ratio ≥1.0 or at least two of the following criteria: 1) right atrial dilatation, 2) dilatation of the pulmonary arteries or 3) reflux of the contrast medium into the hepatic veins and/or the inferior vena cava [3]. RV dysfunction on transthoracic echocardiography (TTE) was defined as RV dilatation (end-diastolic diameter >30 mm from the parasternal view, or a RV/LV diameter ratio ≥1.0 from the subcostal or apical view) combined with right atrial hypertension (absence of inspiratory collapse of the inferior vena cava) in the absence of relevant left ventricular (LV) or mitral valve disease [1].

Tachycardia was defined as heart rate of ≥100 beats per minute (bpm), hypotension as systolic blood pressure <90 mmHg. Hypoxia was defined as a peripheral oxygen saturation <90% or a partial oxygen pressure of <60 mmHg in arterial blood gas analysis. Renal insufficiency was defined as a glomerular filtration rate (GFR) <60 ml/min/1.73 m^2^ body surface area. Active cancer was defined as known disease, treatment with antitumor therapy within the last 6 months, metastatic state or hematologic cancer that was not in complete remission [4]. Major bleeding was defined as fatal bleeding and / or symptomatic bleeding in a critical area or organ (critical bleeding) and / or bleeding causing a fall in haemoglobin level of ≥20 g/l or transfusion of ≥2 units of erythrocyte concentrates (relevant bleeding) according the definition of the *International Society of Thrombosis and Haemostasis* (ISTH) [5].

**Supplementary Results**

*Temporal trends in diagnosis of pulmonary embolism*

Diagnosis of PE was confirmed using CTPA in 92.7%, ventilation / perfusion (V/Q) lung scintigraphy in 6.4% of patients and / or echocardiography (indicating RV failure or showing thrombi in the right heart cavities or proximal pulmonary artery) in haemodynamic unstable patients (1.5%). Usage of the different diagnostic modalities changed over time: While the use of CTPA increased during the study period, V/Q lung scintigraphy was less frequently employed. No significant changes were observed in the frequency of use of TTE in haemodynamically unstable patients (**Table 1s**).

*Reperfusion treatment*

The predominant reperfusion strategy was intravenous systemic thrombolysis (55 patients, 9.1% of all patients). Thrombolysis was administered before hospital admission in 18.2% of cases and in the remaining patients after a median of 2.0 (1.3-4.5) hours after diagnosis. Rescue thrombolysis due to secondary haemodynamic worsening was necessary in two not-high-risk patients. Additionally, 34 (5.6%) patients were included in the Pulmonary Embolism Thrombolysis (PEITHO) trial [6] and thus received double-blind tenecteplase or placebo. Surgical thrombectomy was the primary method in one patient diagnosed with a large intracardiac thrombus. Additionally, four patients underwent rescue surgical thrombectomy after persistent haemodynamic instability despite thrombolysis (n=2) or inclusion in the PEITHO trial (n=2). No patient was treated with an interventional approach.

*Performance of different risk-assessment strategies*

Using different risk assessment strategies, an in-hospital adverse outcome rate of 1.3%, 1.6%, 2.1% and 3.8% were observed in patients classified as low-risk using the European Society of Cardiology (ESC) 2014 algorithm, simplified Pulmonary Embolism Severity Index (sPESI), Bova and modified FAST score, respectively. At the other end of the risk spectrum, patients classified as intermediate-high-risk using the ESC 2014 algorithm, sPESI, Bova and modified FAST score had an in-hospital adverse outcome rate of 12.7%, 8.7%, 13.3% and 13.0%, respectively. As shown in **Table 2s**, the highest sensitivity with regard to an in-hospital adverse outcome was observed for elevation of the cardiac biomarkers troponin and NT-proBNP followed by the sPESI while the highest specificity was observed for the Bova score followed by the modified FAST score and the ESC 2014 algorithm. All risk assessment strategies were able to predict an in-hospital adverse outcome.

**Supplementary Tables**

**Table 1s Trends in use of imaging modalities for diagnosis of PE**

| **Observation period** | **09/2008-08/2016**  **(n=605)** | **09/2008-08/2010**  **(n=145)** | **09/2010-08/2012**  **(n=140)** | **09/2012-08/2014**  **(n=165)** | **09/2014-08/2016**  **(n=155)** | **P for trend** |
| --- | --- | --- | --- | --- | --- | --- |
| CTPA | 561/605 (92.7%) | 122/145 (84.1%) | 138/140 (98.5%) | 154/165 (93.3%) | 147/155 (94.8%) | **0.005** |
| V/Q lung scintigraphy | 39/605 (6.4%) | 19/145 (13.1%) | 3/140 (2.1%) | 10/165 (6.7%) | 7/155 (4.5%) | **0.016** |
| TTE in haemodynamic unstable patients | 9/605 (1.5%) | 5/145 (3.4%) | 0/140 (0%) | 2/165 (1.2%) | 2/155 (1.3%) | 0.24 |

Abbreviations: PE: pulmonary embolism; CTPA: computed tomographic pulmonary angiography; V/Q: ventilation / perfusion; TTE: transthoracic echocardiography

**Table 2s Prognostic performance of risk assessment strategies in not-high risk patients**

|  | **AUC  [95% CI]** | **Cut-off value** | **Sensitivity [95% CI]** | **Specificity [95% CI]** | **PPV  [95% CI]** | **NPV  [95% CI]** | **PLR  [95% CI]** | **NLR  [95% CI]** | **OR [95% CI],  p-value** |
| --- | --- | --- | --- | --- | --- | --- | --- | --- | --- |
| **A: In-hospital adverse outcome** |  |  |  |  |  |  |  |  |  |
| ESC 2014 algorithm | 0.68  [0.59-0.77] | intermediate-high risk | 0.60  [0.42-0.76] | 0.72  [0.68-0.76] | 0.12  [0.08-0.19] | 0.96  [0.94-0.98] | 2.1  [1.6-2.9] | 0.6  [0.4-0.8] | **3.8 [1.9-7.7], p<0.001** |
| sPESI | 0.71  [0.63-0.79] | ≥1 point(s) | 0.91  [0.76-0.98] | 0.35  [0.31-0.39] | 0.09  [0.06-0.12] | 0.98  [0.95-1.0] | 1.4  [1.2-1.6] | 0.2  [0.1-0.7] | **5.7 [1.7-19.0], p=0.004** |
| Bova score | 0.70  [0.62-0.77] | ≥5 points | 0.31  [0.17-0.49] | 0.86  [0.83-0.89] | 0.13  [0.07-0.23] | 0.95  [0.92-0.97] | 2.2  [1.3-3.8] | 0.8  [0.6-1.0] | **2.8 [1.3-6.0], p<0.007** |
| modified FAST score | 0.68  [0.59-0.77] | ≥3 points | 0.57  [0.39-0.73] | 0.74  [0.70-0.78] | 0.13  [0.08-0.20] | 0.96  [0.94-0.98] | 2.2  [1.6-3.0] | 0.6  [0.4-0.9] | **3.8 [1.9-7.6], p<0.001** |
| Tachycardia | 0.61  [0.50-0.73] | HR ≥100/min | 0.53  [0.35-0.70] | 0.66  [0.62-0.71] | 0.10  [0.06-0.15] | 0.95  [0.93-0.97] | 1. 6 [1.1-2.2] | 0.7  [0.5-1.0] | **2.2 [1.1-4.5], p=0.023** |
| Troponin | 0.73  [0.66-0.80] | ≥14 pg/ml (hsTnT) or  ≥13/33 ng/ml (cTnI)^#^ | 1.0  [0.85-1.0] | 0.36  [0.32-0.41] | 0.09  [0.06-0.13] | 1.0  [0.97-1.0] | 1.6  [1.5-1.7] | n.c.^a^ | n.c.^a^ |
| NT-proBNP | 0.78  [0.72-0.84] | ≥600 pg/ml | 1.0  [0.84-1.0] | 0.51  [0.46-0.56] | 0.12  [0.08-0.17] | 1.0  [0.98-1-00] | 2.0  [1.8-2.2] | n.c.^a^ | n.c.^a^ |
| RV dysfunction on TTE / CT |  |  | 0.73  [0.54-0.87] | 0.53  [0.48-0.57] | 0.09  [0.06-0.14] | 0.97  [0.94-0.99] | 1.5  [1.2-2.0] | 0.5  [0.3-0.9] | **3.0 [1.3-7.0], p=0.008** |
| **B: In-hospital all-cause mortality** |  |  |  |  |  |  |  |  |  |
| ESC 2014 algorithm | 0.64  [0.52-0.75] | intermediate-high risk | 0.52  [0.31-0.73] | 0.71  [0.67-0.75] | 0.07  [0.04-0.13] | 0.97  [0.95-0.98] | 1.8  [1.2-2.7] | 0.7  [0.5-1.0] | **2.6 [1.1-6.1], p=0.024** |
| sPESI | 0.69  [0.59-0.80 | ≥1 point(s) | 0.91  [0.70-0.98] | 0.34  [0.30-0.39] | 0.06  [0.04-0.09] | 0.99  [0.96-1.00] | 1.4  [1.2-1.6] | 0.3  [0.1-1.0] | **5.5 [1.3-23.8], p=0.022** |
| Bova score | 0.63  [0.54-0.72] | ≥5 points | 0.17  [0.06-0.40] | 0.85  [0.82-0.88] | 0.05  [0.02-0.13] | 0.96  [0.94-0.98] | 1.2  [0.5-2.9] | 1.0  [0.8-1.2] | 1.1 [0.4-3.6], p=0.76 |
| modified FAST score | 0.60  [0.48-0.73] | ≥3 points | 0.48  [0.27-0.69] | 0.73  [0.69-0.77] | 0.07  [0.04-0.13] | 0.97  [0.95-0.98] | 1.8  [1.2-2.8] | 0.7  [0.5-1.1] | **2.5 [1.1-5.7], p=0.036** |
| Tachycardia | 0.60  [0.47-0.72] | HR ≥100/min | 0.50  [0.29-0.71] | 0.66  [0.62-0.70] | 0.06  [0.03-0.11] | 0.97  [0.94-0.98] | 1.5  [1.0-2.3] | 0.8  [0.5-1.2] | 1.9 [0.8-4.6], p=0.13 |
| Troponin^b^ | 0.64 [0.53-0.74] | ≥14 pg/ml (hsTnT) or  ≥13/33 ng/ml (cTnI)^c^ | 0.88  [0.62-0.98] | 0.35  [0.31-0.40] | 0.05  [0.03-0.08] | 0.99  [0.95-1.0] | 1.4  [1.1-1.6] | 0.3  [0.1-1.2] | 4.1 [0.9-18.0], p=0.06 |
| NT-proBNP | 0.68  [0.58-0.78] | ≥600 pg/ml | 0.87  [0.58-0.98] | 0.49  [0.44-0.54] | 0.06  [0.03-0.10] | 0.99  [0.96-1.00] | 1.7  [1.4-2.1] | 0.3  [0.1-1.0] | **6.2 [1.4-27.8], p=0.017** |
| RV dysfunction on TTE / CT |  |  | 0.67 [0.41-0.86] | 0.52  [0.47-0.56] | 0.05  [0.03-0.09] | 0.98 [0.95-0.99] | 1.4  [1.0-1.9] | 0.6 [0.3-1.2] | 2.1 [0.8-5.8], p=0.14 |

^a^ Could not be calculated due to a sensitivity of 1.00 of the respective parameters. ^b^ AUC is provided for hsTnT only. ^c^ for female/male patients

Abbreviations: AUC: area under the curve; CI: confidence interval; PPV: positive predictive value; NPV: negative predictive value; PLR: positive likelihodd ratio; NLR: negative likelihood ratio; OR: odds ratio; ESC: European Society of Cardiology; sPESI: simplified Pulmonary Embolism Severity Index; HR: heart rate; NT-proBNP: n.c.: not calculable; N-terminal pro-brain natriuretic peptide; RV: right ventricular; TTE: transthoracic echocardiography; CT: computed tomography

**Table 3s Prognostic performance of risk assessment strategies in not-high risk patients over time**

| **Observation period** |  | **09/2008-08/2012** | | | **09/2012-08/2016** | | |
| --- | --- | --- | --- | --- | --- | --- | --- |
| **In-hospital adverse outcome** |  | **18/258 (7.0%)** | | | **17/292 (5.8%)** | | |
|  | **Cut-off value** | **Sensitivity  [95% CI]** | **Specificity  [95% CI]** | **OR [95% CI],  p-value** | **Sensitivity  [95% CI]** | **Specificity  [95% CI]** | **OR [95% CI],  p-value** |
| ESC 2014 algorithm | intermediate-high risk | 0.67  [0.41-0.86] | 0.64  [0.58-0.70] | **3.6 [1.3-9.9], p=0.001** | 0.53  [0.29-.76] | 0.79  [0.73-0.83] | **4.1 [1.5-11.1], p=0.005** |
| sPESI | ≥1 point(s) | 0.89  [0.64-0.98] | 0.32  [0.26-0.38] | 3.7 [0.8-16.5], p=0.09 | 0.94  [0.69-1.00] | 0.38  [0.32-0.44] | **9.9 [1.3-75.6], p=0.027** |
| Bova score | ≥5 points | 0.44  [0.22-0.69] | 0.83  [0.77-0.87] | **3.8 [1.4-10.1], p=0.009** | 0.18  [0.05-044] | 0.89  [0.85-0.92] | 1.8 [0.5-6.4], p=0.40 |
| modified FAST score | ≥3 points | 0.61  [0.36-0.82] | 0.72  [0.65-0.77] | **4.0 [1.5-10.7], p=0.006** | 0.53  [0.29-0.76] | 0.76  [0.70-0.81] | **3.6 [1.3-9.6], p=0.012** |
| Tachycardia | HR ≥100/min | 0.50  [0.27-0.73] | 0.67  [0.60-0.73] | 2.0 [0.8-5.2], p=0.16 | 0.56  [0.31-0.79] | 0.67  [0.61-0.72] | 2.6 [0.9-7.1], p=0.07 |
| Troponin | ≥14 pg/ml (hsTnT) or  ≥13/33 ng/ml (cTnI)^#^ | 1.00 [0.76-1.00] | 0.35  [0.29-0.41] | n.c.^a^ | 1.00 [0.70-1.00] | 0.38  [0.32-0.45] | n.c.* |
| NT-proBNP | ≥600 pg/ml | 1.00  [0.77-1.00] | 0.48  [0.41-0.55] | n.c.^a^ | 1.00  [0.63-1.00] | 0.54  [0.47-0.62] | n.c.* |
| RV dysfunction on TTE / CT |  | 0.72  [0.46-0.89] | 0.47  [0.41-0.54] | 2.3 [0.8-6.7], p=0.12 | 0.75  [0.43-0.93] | 0.58  [0.51-0.64] | **4.1 [1.1-15.7], p=0.037** |

^a^ Could not be calculated due to a sensitivity of 1.00 of the respective parameters

Abbreviations: CI: confidence interval; OR: odds ratio; ESC: European Society of Cardiology; sPESI: simplified Pulmonary Embolism Severity Index; n.c.: not calculable; NT-proBNP: N-terminal pro-brain natriuretic peptide; RV: right ventricular; TTE: transthoracic echocardiography; CT: computed tomography

**Table 4s Differences in patients’ baseline characteristics with regard to survival status after one-year**

|  | **Overall survivors (n=503)** | **In-hospital death (n=44)** | **P-value** | **Death after discharge (n=58)** | **P-value** | **Overall decedents (n=102)** | **P-value** |
| --- | --- | --- | --- | --- | --- | --- | --- |
| Age (years) | 69 [54-77] | 74 [59-79] | 0.05 | 73 [65-79] | **0.007** | 73 [61-79] | **0.002** |
| Sex (female) | 53.3% | 47.7% | 0.48 | 53.4% | 0.98 | 51.0% | 0.67 |
| BMI (kg/m²) | 27.7 [24.5-31.6] | 26.8 [23.4-30.9] | 0.36 | 26.7 [23.4-30.2] | 0.84 | 26.8 [23.8-30.6] | 0.06 |
| **Risk factors for VTE and comorbidities** |  |  |  |  |  |  |  |
| Previous VTE | 145/502 (28.9%) | 7/43 (16.3%) | 0.07 | 9/58 (15.5%) | **0.031** | 16/101 (15.8%) | **0.007** |
| Surgery (previous 4 weeks) | 85/503 (16.9%) | 13/44 (29.5%) | **0.036** | 11/58 (19.0%) | 0.69 | 24/102 (23.5%) | 0.11 |
| Trauma (previous 4 weeks) | 17/503 (3.4%) | 1/44 (2.3%) | 0.69 | 0/58 (0%) | 0.24 | 1/102 (1.0%) | 0.19 |
| Immobilisation (previous 4 weeks) | 97/503 (19.3%) | 13/43 (30.2%) | 0.08 | 17/58 (29.3%) | 0.07 | 30/101 (29.7%) | **0.019** |
| Travel | 30/502 (6.0%) | 0/43 (0%) | 0.10 | 1/58 (1.7%) | 0.18 | 1/101 (1.0%) | **0.038** |
| Cancer | 51/503 (10.1%) | 16/44 (36.4%) | **<0.001** | 35/58 (60.3%) | **<0.001** | 51/102 (50.0%) | **<0.001** |
| Chronic heart failure | 77/503 (15.3%) | 6/44 (13.6%) | 0.77 | 13/58 (22.4%) | 0.16 | 19/102 (18.6%) | 0.40 |
| Chronic pulmonary disease | 72/503 (14.3%) | 8/44 (18.2%) | 0.49 | 13/58 (22.4%) | 0.10 | 21/102 (20.6%) | 0.11 |
| Renal insufficiency | 164/491 (40.0%) | 24/40 (60.0%) | **0.001** | 19/58 (32.8%) | 0.92 | 43/98 (43.9%) | **0.047** |
| **Pre-existing anticoagulant treatment** |  |  |  |  |  |  |  |
| Therapeutic anticoagulation | 26/503 (5.2%) | 4/43 (9.3%) | 0.28 | 5/58 (8.6%) | 0.36 | 9/101 (8.9%) | 0.14 |
| Prophylactic anticoagulation | 39/503 (7.8%) | 7/43 (16.3%) | 0.05 | 6/58 (10.3%) | 0.49 | 13/101 (12.9%) | 0.09 |
| **Symptoms and clinical findings** |  |  |  |  |  |  |  |
| Chest pain | 261/503 (51.9%) | 12/42 (28.6%) | **0.004** | 19/57 (33.3%) | **0.008** | 31/99 (31.3%) | **<0.001** |
| Dyspnoea | 416/503 (82.7%) | 31/42 (73.8%) | 0.15 | 49/57 (86.0%) | 0.53 | 80/99 (80.1%) | 0.65 |
| Haemoptysis | 17/503 (3.4%) | 0/42 (0%) | 0.23 | 1/58 (1.7%) | 0.50 | 1/100 (1.0%) | 0.20 |
| Syncope | 74/503 (14.7%) | 13/43 (30.2%) | **0.008** | 7/58 (12.1%) | 0.59 | 20/101 (19.8%) | 0.20 |
| Unilateral leg swelling | 112/501 (22.4%) | 8/42 (19.0%) | 0.62 | 18/57 (31.6%) | 0.12 | 26/99 (26.3%) | 0.40 |
| Tachycardia | 165/489 (33.7%) | 16/36 (44.4%) | 0.19 | 27/56 (48.2%) | **0.032** | 43/92 (46.7%) | **0.017** |
| Hypotension | 18/482 (3.7%) | 10/32 (31.3%) | **<0.001** | 6/56 (10.7%) | **0.017** | 16/88 (18.2%) | **<0.001** |
| Hypoxia | 105/426 (24.6%) | 25/39 (64.1%) | **<0.001** | 17/46 (37.0%) | 0.07 | 42/85 (49.4%) | **<0.001** |
| RV dysfunction on TTE / CT | 227/459 (49.5%) | 28/36 (77.8%) | **0.001** | 29/54 (53.7%) | 0.56 | 57/90 (63.3%) | **0.016** |
| Elevated troponin | 284/447 (63.5%) | 32/35 (91.4%) | **0.001** | 43/49 (87.8%) | **0.001** | 75/84 (89.2%) | **<0.001** |
| Elevated NT-proBNP | 196/392 (50.0%) | 18/25 (72.0%) | **0.033** | 30/41 (73.2%) | **0.005** | 48/66 (72.7%) | **0.001** |

All p-values were calculated comparing patients in the respective columns to survivors (n=503)

Abbreviations: BMI: body mass index; VTE: venous thromboembolism; RV: right ventricular; TTE: transthoracic echocardiography; CT: computed tomography; NT-proBNP: N-terminal pro-brain natriuretic peptide

**Table 5s Prognostic performance of risk markers and assessment strategies with regard to in-hospital and one-year mortality**

|  | **In-hospital death (44/547)***  **OR [95% CI]** | **Death after discharge (58/561)#**  **OR [95% CI]** | **Overall decedents (102/605)**  **OR [95% CI]** |
| --- | --- | --- | --- |
| ESC 2014 low-risk | **0.13 [0.02-0.92], p=0.041** | not calculable | **0.05 [0.01-0.39], p=0.004** |
| ESC 2014 high-risk | **16.8 [8.2-34.1], p<0.001** | **2.9 [1.3-6.8], p=0.012** | **7.3 [4.1-13.1], p<0.001** |
| sPESI ≥1 point(s) | **7.7 [2.3-25.2], p=0.001** | **32.0 [4.4-233.3], p=0.001** | **13.8 [5.0-38.0], p<0.001** |
| Bova score ≥5 points | 1.5 [0.6-3.8], p=0.40 | 1.8 [0.9-3.7], p=0.09 | 1.7 [1.0-3.0], p=0.07 |
| modified FAST score ≥3 points | 1.8 [1.0-3.6], p=0.05 | 1.5 [0.8-2.6], p=0.17 | **1.6 [1.1-2.6], p=0.028** |
| Tachycardia | 1.6 [0.8-3.1], p=0.20 | **1.8 [1.1-3.2], p=0.034** | **1.7 [1.1-2.7], p=0.018** |
| Troponin elevation | **6.1 [1.8-20.3], p=0.003** | **4.1 [1.7-9.9], p=0.002** | **4.7 [2.3-9.8], p<0.001** |
| NT-proBNP elevation | **2.6 [1.1-6.3], p=0.039** | **2.7 [1.3-5.6], p=0.006** | **2.7 [1.5-4.7], p=0.001** |
| RV dysfunction on TTE / CT | **3.6 [1.6-8.0], p=0.002** | 1.2 [0.7-2.1], p=0.56 | **1.8 [1.1-2.8], p=0.017** |

* patients who died after discharge (n=58) were excluded from analyses

# patients who died during the in-hospital stay (n=44) were excluded from analyses

Abbreviations: OR: odds ratio; CI: confidence interval; ESC: European Society of Cardiology; sPESI: simplified Pulmonary Embolism Severity Index; NT-proBNP: N-terminal pro-brain natriuretic peptide; RV: right ventricular; TTE: transthoracic echocardiography; CT: computed tomography

**Supplementary References**

1. Lankeit M, Friesen D, Aschoff J, Dellas C, Hasenfuss G, Katus H, Konstantinides S, Giannitsis E (2010) Highly sensitive troponin T assay in normotensive patients with acute pulmonary embolism. Eur Heart J 31 (15):1836-1844. doi:10.1093/eurheartj/ehq234

2. Lankeit M, Jimenez D, Kostrubiec M, Dellas C, Kuhnert K, Hasenfuss G, Pruszczyk P, Konstantinides S (2014) Validation of N-terminal pro-brain natriuretic peptide cut-off values for risk stratification of pulmonary embolism. Eur Respir J 43 (6):1669-1677. doi:10.1183/09031936.00211613

3. Hellenkamp K, Schwung J, Rossmann H, Kaeberich A, Wachter R, Hasenfuss G, Konstantinides S, Lankeit M (2015) Risk stratification of normotensive pulmonary embolism: prognostic impact of copeptin. Eur Respir J 46 (6):1701-1710. doi:10.1183/13993003.00857-2015

4. Raskob GE, van Es N, Segers A, Angchaisuksiri P, Oh D, Boda Z et al. (2016) Edoxaban for venous thromboembolism in patients with cancer: results from a non-inferiority subgroup analysis of the Hokusai-VTE randomised, double-blind, double-dummy trial. Lancet Haematol 3 (8):e379-387. doi:10.1016/S2352-3026(16)30057-6

5. Schulman S, Kearon C, Subcommittee on Control of Anticoagulation of the S, Standardization Committee of the International Society on T, Haemostasis (2005) Definition of major bleeding in clinical investigations of antihemostatic medicinal products in non-surgical patients. J Thromb Haemost 3 (4):692-694. doi:10.1111/j.1538-7836.2005.01204.x

6. Meyer G, Vicaut E, Danays T, Agnelli G, Becattini C, Beyer-Westendorf J et al. (2014) Fibrinolysis for patients with intermediate-risk pulmonary embolism. N Engl J Med 370 (15):1402-1411. doi:10.1056/NEJMoa1302097
